# Cost of breast cancer based on real-world data: a cancer registry study in Italy

**DOI:** 10.1186/s12913-017-2006-9

**Published:** 2017-01-26

**Authors:** Stefano Capri, Antonio Russo

**Affiliations:** 1 School of Economics and Business, Cattaneo-LIUC University, Corso Matteotti 22, 21053 Castellanza, VA Italy; 2 Epidemiology Unit, Agency for Health Protection of the Province of Milan, C.so Italia 19, 20122 Milan, Italy

**Keywords:** Breast cancer, Costs, Real world data, Health care utilization, Italy

## Abstract

**Background:**

In European countries, it is difficult for local health organizations to determine the resources allocated to different hospitals for breast cancer. The aim of the current study was to examine the costs of breast cancer during the different phases of the diagnostictherapeutic sequence based on real world data.

**Methods:**

To identify breast cancer cases diagnosed between 2007 and 2011, we used the cancer registry of the Agency for Health Protection of the Province of Milan (3.2 million inhabitants). A generalized linear model controlling for patient age, cancer stage and Charlson co-morbidity index was used to calculate the adjusted mean costs for each hospital and for each study phase. Regression analyses were based on dependent variables of individual costs (diagnosis, treatment, follow-up and total cost were logtransformed. The following independent variables were included as covariates: age at diagnosis, hospital volume, stage, job category, educational level, marital status, comorbidities, deprivation index. Total and mean costs were computed for several variables and for each phase. On average for each subject, the costs were collected over 2.5 years.

**Results:**

A total of 12,580 breast cancer cases were studied. The mean cost of diagnosis was €414, the mean cost of treatment was €8,780, the mean overall cost of follow-up was approximately €2,351, and the mean total direct medical cost was €10,970. The age of the patients, stage of tumor and employment level of the patient were significantly correlated with the variability of the costs. The highest variability in costs was observed for the follow-up costs, in which 38% of hospitals fell outside the 95% confidence interval. In the overspending-hospitals, patients received an intensive follow-up regimen with scintigraphy and thoracic CAT (computerized axial tomography).

**Conclusions:**

In this study, which represents the first population-level study of its kind in Italy, we estimated all direct medical costs for the 6-month period before the diagnosis of breast cancer and the first two years after diagnosis. Patients were identified from the local cancer registry. The analysis offers insight into the utilization of resources incurred by one major area of interest of cancer care in Italy.

## Background

The guidelines for the diagnosis, treatment and follow-up of breast cancer patients are well defined. Over the last decades, a number of diagnostic procedures, treatments and follow-up programs have been introduced, significantly improving patient outcomes. However, there remains a lack of information and evidence concerning the management of this disease based on real-world data. It is particularly difficult for healthcare organizations, notably those in European countries, to determine how resources are allocated to different hospitals during the different phases of care for the disease.

Many studies have estimated the economic burden of breast cancer [1–3]. Across the EU countries, breast cancer accounts for the highest health-care costs, followed by colorectal cancer, prostate cancer and lung cancer [1].

However, the estimates of the mean costs for inhabitants, although informative for projecting the total healthcare expenditure and for comparing different healthcare systems, are not very useful for decision makers who need to evaluate the variability in resources utilization.

Health policies in high-income countries must incorporate a comprehensive estimation of the costs of cancer care to provide appropriate and economically affordable cancer care [4, 5].

The purpose of this study was to achieve a better understanding of the distribution of costs during the different phases of care for the disease [6, 7] according to the age of the patients and in relation to the hospital volumes. In particular, our objectives were as follows: 1) to estimate the cost of breast cancer during the first 2.5 years after the diagnosis, 2) to determine in which phase of the diagnostic-therapeutic sequence most of the costs are incurred, 3) to better understand how variables influence the average cost and 4) to assess the correlation between complexity measured by the volume of breast cancer treated (size of the hospital) and the variability of costs.

Finally, like every cost analysis in healthcare [8] concerning the overall utilization and attributable costs associated with breast cancer care, this study intends to inform decision makers. Attributable costs refer to those costs incurred by patients that are thought to be directly related to the detection or treatment of the disease [9].

The use of cancer registry data offers the key advantage of more accurately identifying the incident cases and of studying defined cohorts [10, 11].

The extended use of the administrative data systems, particularly through linkage to other datasets, will provide vital support to the development of national health policies.

Policymakers depend on such studies to inform decisions on healthcare financing.

## Methods

### Setting

We conducted a retrospective descriptive study using the health information systems of the Agency for Health Protection of the Province of Milan (3.2 million inhabitants), the largest metropolitan area of the Northern of Italy.

Patients were identified using the local cancer registry based on a validated automated methodology that uses clinical sources of information (archive of death certificates, hospital discharge summaries and histo-cytopathological reports) with an efficient system of record linkage and algorithm recognition to match all data at the individual level.

All breast cancer cases diagnosed between Jan.1, 2007 and Dec. 31, 2011 (ICD-O3 topographic code C50) except extranodal lymphomas and sarcomas were included in this study. For each breast cancer case, information concerning sociodemographic characteristics, deprivation index, tumor morphology, staging (TNM) and grading were available. The cancer stage was collected by the Cancer Registry, according to the cancer registration rules, at diagnosis: M1 stage was assigned considering all distant diseases diagnosed between the cancer incidence and 6 months after.

To estimate the costs, the profiles for each case were identified through access to the Health Care Information System beginning 6 months prior to diagnosis and through the two subsequent years.

All care utilization from January 2006 to December 2014 were extracted from the Health Information System of the Province of Milan of the following databases: (1) the Pharmaceutical Prescriptions Registry (209 million prescriptions), (2) the Hospital Discharge Registry (7 million admissions), and the Outpatients Registry (538 million prescriptions).

These three sources were linked to each other at an individual level for each cancer case included in the study period using the Italian fiscal code as a unique identifier. These sources covered all direct medical costs attributable to the Italian National Health Service.

Only the direct medical costs paid by patients (out of pocket) were excluded. It is worth noting that in Italy, the public healthcare system covers almost all direct medical costs for cancer patients.

#### Pharmaceutical prescriptions

Pharmaceutical prescriptions were coded according to the Anatomical Therapeutic Chemicals (ATC) classification. All prescriptions of antineoplastic drugs (ATC code: L01, L02, L03) were included in the study. Costs were derived from the prices of each specific authorization number issued by the Italian Drugs Agency for commercialization (AIC). In addition, the utilization of biological drugs was included according the separate budget in which they were reported, referring to the Regional Health Authority and not to the individual hospital.

#### Hospital discharges

All hospital admission related to cancer care were included in the study. The selection was based on the specific ICD-IX CM procedure codes, including cytology/biopsy, specific surgery, chemotherapy, radiotherapy, laboratory analysis, and imaging. Each hospital stay was also identified according to the Diagnosis-Related Groups (DRGs) codes.

#### Outpatient care

The outpatient visits were divided into 38 specialties. To assess cancer-related financial costs, sub-categorizations were created: oncology, radiotherapy, laboratory analysis, imaging studies and other clinical investigations.

#### Deprivation index

The Deprivation index was derived from the 2001 General Census of Population and Housing. From the 280 variables defined at the census block level, low level of education, unemployment, lack of home ownership, single-parent family and overcrowding were integrated by summing standardized indicators in the form of a deprivation index. Quintiles of the indicator distribution were used. Deprivation index was calculated at residence level and to all subject resident in the same municipality was attributed a unique value of deprivation index [12].

### Estimation of costs

According to an updated methodology, we used a phase-based approach. The period between 6 months prior to diagnosis and the two subsequent years was subdivided into three different phases: 1) the diagnostic phase from 6 months before diagnosis or surgery, 2) the treatment phase from diagnosis or surgery through 12 months after diagnosis/surgery, and 3) the follow-up phase from 3 months after diagnosis or surgery to 24 months after diagnosis/surgery.

The overlap between the various phases was limited by the identification of specific procedure codes for every phase (e.g., chemotherapy is allocated only in the phase of treatment but not in the follow-up stage). However, each access was considered only once in a specific phase.

For each subject and for each phase, only the costs attributable to breast cancer were calculated by summing the drug, outpatient and hospital costs related to breast cancer. However, considering that the DRGs reimbursement system is a reimbursement system based on fixed tariffs, some general activities, e.g., Emergency Room (ER), Intensive Care Unit (ICU), and approximately 20% of the final annual hospital budget was not financed by DRGs activities. To allow the estimation costs to be more accurate and to take into account the differences among hospitals (e.g., some hospital in the study did not have an ER or an ICU), the annual costs were divided by the total number of hospital days provided for all admissions during the year. Therefore, a daily correction factor specific for each hospital was calculated and added to the DRG by multiplying the daily value (in euros) by the number of hospital days. All costs were reported in 2014 euros. Normally, the costs as cumulative sums are adjusted to constant euros corresponding to a given calendar year [13]. The last year of observation was 2014 (the reference year). However, the objective of this study was to account for the variability of costs related to many variables rather than to estimate the general cost for a specific cancer; therefore, it seemed more suitable to attribute the cost value in the more recent unit costs available, i.e., 2014 euros (the current prices and tariffs applied in the Province of Milan).

#### Statistical analysis

Total and mean costs were computed for several variables and for each phase. On average for each subject, the costs were collected over 2.5 years.

The information was summarized by creating synthetic graphics. A generalized linear model (GLM) was constructed controlling for patient age, stage and Charlson index [14] to calculate the adjusted mean costs for each hospital and for each study phase. Funnel plots, a type of scatter plot of the effect measure against a measure of the study size, were used to visualize the relationship between the adjusted mean costs and the hospital volume (defined as the mean number of radical or conservative breast cancer surgical procedures between 2006–2011) [15, 16].

We identified the major predictors of breast cancer variability cost by using a general linear model (GLM). Dependent variables of individual costs: diagnosis, treatment, follow-up and total cost were log-transformed. The following independent variables were included as covariates: age at diagnosis, hospital volume, stage, job category, educational level, marital status, comorbidities, deprivation index.

All analyses were performed using SAS 9.4 [17].

## Results

Our study cohort consisted of 12,580 patients who were diagnosed with breast cancer. Their demographic information is summarized in Table 1 (The three categories of age (<45, 45–69, >69) are the major categories of mammography screening to identify women be invited in Italy).

**Table 1:** Characteristics of 12.580 incident cases of breast cancer (2007–2011) and adjusted means of care costs (diagnosis, treatment, follow-up and overall; €)

	N.	%	Costs (€)			
			diagnosis	treatment	follow-up	overall
Age at diagnosis						
<45	1,390	*11.05*	296.77	10,000.83	2,543.42	12,210.85
45–69	6,913	*54.95*	368.91	7,832.36	2,308.19	9,841.23
69+	4,277	*34.00*	539.57	4,949.00	2,064.76	6,692.16
Hospital type						
Cancer Center	6,058	*48.16*	758.55	9,552.09	3,027.95	12,522.18
Public general hospital	4,464	*35.48*	846.24	8,705.54	2,798.75	11,416.47
Private general hospital	1,406	*11.18*	709.07	7,986.39	2,592.26	10,466.91
Country hospital	8,114	*5.18*	61.9	4,132.24	802.86	4,620.09
Hospital volume						
low (<50)	1,570	*12.48*	491.33	491.33	2,459.34	9,206.66
medium (50–150)	2,896	*23.02*	535.07	7,711.82	2,464.23	9,842.53
high (>150)	2,174	*64.50*	372.96	7,690.33	2,254.35	9,646.24
Stage						
T1N0M0	4,641	*36.89*	144.1	7,277.0	1,783.4	9,014.8
T2-3N0M0	1,350	*10.73*	197.1	8,702.8	2,179.5	10,755.8
T1-3 N + M0	3,872	*30.78*	201.1	10,937.0	2,594.7	13,435.9
T4M0	1,188	*9.44*	237.8	8,828.7	2,403.7	11,175.1
M1	1,108	*8.81*	2,261.9	9,177.8	4,575.6	12,825.9
Not available	421	*3.35*	1,427.5	2,376.6	2,562.0	4,577.9
Employment category						
Professional/clerical	3,024	*24.04*	372.51	8,570.82	2,759.54	11,045.03
Manual workers	717	*5.70*	334.60	8,580.01	2,665.97	10,914.96
Housewife	2,126	*16.90*	383.43	7,827.24	2,732.78	10,226.13
Pensioner	5,081	*40.39*	368.28	7,147.76	2,189.88	8,970.62
Other	1,115	*8.86*	392.25	8,062.96	1,772.34	9,510.34
Missing	517	*4.11*	672.27	5,273.02	1,238.66	6,427.42
Educational level						
Elementary school	2,304	*18.31*	402.06	7,959.91	2,347.13	10,010.70
Junior high school	5,599	*44.51*	378.09	7,457.62	2,254.22	9,355.50
High school	2,455	*19.52*	335.90	8,281.07	2,767.98	10,720.93
College or higher	1,713	*13.62*	336.62	8,207.02	2,513.18	10,420.52
Missing	509	*4.05*	723.22	5,361.00	1,254.54	6,560.85
Marital status						
Living alone	1,580	*12.56*	344.76	7,151.48	2,074.98	8,889.80
Married	7,132	*56.69*	375.55	7,718.56	2,400.29	9,831.68
Divorced	497	*3.95*	361.05	7,443.40	2,760.63	9,948.02
Widowed	2,641	*20.99*	443.45	7,111.70	2,223.60	8,998.46
Missing	730	*5.80*	637.55	9,182.65	2,002.38	10,800.96
Deprivation Index (quintiles)						
I	2,516	*20.00*	417.76	7,806.33	2,278.88	9,744.27
II	2,614	*20.78*	457.46	7,187.08	2,078.35	8,955.19
III	2,418	*19.22*	422.28	7,449.98	2,248.47	9,395.27
IV	2,445	*19.44*	369.28	7,695.16	2,572.96	9,956.93
V	2,587	*20.56*	340.08	7,979.30	2,409.76	10,025.87
Co-morbidities						
None	7,421	*58.99*	419.96	7,785.99	2,140.01	9,605.12
1	3,049	*24.24*	391.94	7,340.02	2,186.26	9,204.86
2	1,526	*12.13*	369.89	7,223.88	2,613.24	9,549.07
3+	584	*4.64*	354.67	7,357.32	3,967.09	10,931.86
Overall	12,580		414.7	8,780.2	2,351.7	10,970.1

The mean diagnosis cost was €414, ranging from the minimum of €144 for stage T + N0M0 to the maximum of €2,261 for stage M1. The mean treatment cost was €8,780, approximately 80% of the total cost. With respect to the mean treatment cost, patients <45 years of age incurred higher mean treatment costs (€10,000), and patients >69 years of age incurred lower mean treatment costs (€4,949). Only 11% of the patients (1,393) were treated with expensive biologics. The mean overall follow-up cost was approximately €2,351, ranging from the minimum of €1,783 for stage T + N0M0 and the maximum for stage M1 (€4,575).

Finally, the mean total direct medical cost was €10,970, with minimum costs reported for patients >69 years of age (€6,692) and maximum costs reported for metastatic patients (M1) (€12,825) and for patients <45 years of age (€12,210).

The distribution on cost items showed that hospital admission account for a 71% of total cost (mean cost €8,242.00) and the outpatients procedures 14% (€1,634.00, mainly radiology) and the chemotherapy drugs 15% (1,811.00).

As is widely recognized, most empirical analyses of healthcare cost data are regressionbased [18, 19]. We performed regression analyses followed by funnel plot analyses.

The regression analyses revealed that cancer stage was associated with a significantly increased overall cost and treatment cost (Table 2). The age of the patient was significantly correlated with lower costs for treatment, follow-up and overall costs. The employment status of the patient also significantly influenced all variable costs, resulting in higher expenditures for higher levels of patient employment. Co-morbidities accounted for a lower reduction in the diagnosis cost.

**Table 2:** Multiple regression models for different variables using as dependent variable individual cost for diagnosis, treatment, follow up and overall

	diagnosis		treatment		follow up		overall	
	F value	Pr > F	F value	Pr > F	F value	Pr > F	F value	Pr > F
Age at diagnosis	1.01	0.3657	130.53	<.0001	26.89	<.0001	89.19	<.0001
Hospital type	35.98	<.0001	142.17	<.0001	42.33	<.0001	440.53	<.0001
Hospital volume	0.00	0.9963	1.55	0.2124	12.61	<.0001	0.65	0.5245
Stage	153.32	<.0001	287.52	<.0001	47.50	<.0001	115.46	<.0001
Employment category	0.60	0.7007	2.17	0.0544	5.22	<.0001	5.03	0.0001
Educational level	0.68	0.6044	0.57	0.6843	3.48	0.0075	1.86	0.1137
Marital status	4.58	0.0011	5.55	0.0002	3.73	3.73	9.82	<.0001
Deprivation Index	1.91	0.1056	4.63	4.63	6.72	<.0001	6.57	<.0001
Comorbidities	1.21	0.3056	5.61	0.0008	36.16	<.0001	5.85	0.0005
*R-square*	*R-square*		*0.29*		*0.70*		*0.33*	

**Fig. 1:**
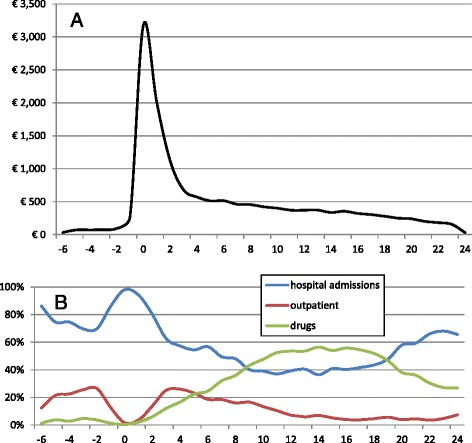
Total cost of care (**a**) and (**b**) proportional cost for hospital admission, outpatient visits and drugs from 6 months prior to 24 months after diagnosis (€)

We examined the trends for the total costs during the 30-month period surrounding diagnosis for each patient (Fig. 1). Not surprisingly, the costs were higher in the 1.5-month interval before surgery and the 1.5–month interval following surgery, during which patients were hospitalized not only for their surgical intervention but also for staging of the tumor and for occasional post-surgical complications (Fig. 1a). The cost of drugs increased sharply with the reduction in the hospital admission costs, representing the chemotherapy cycles (Fig. 1b). Before the hospitalization, the costs of drugs consisted of neoadjuvant chemotherapy; after the hospitalization, these costs were due to the adjuvant chemotherapy. The resources used in the outpatient setting within a two-month window before and after the hospital stay involved radiation therapies, which were prescribed before the surgery for some patient and after for some others, as well as other visits.

The mean total cost was higher for younger patients than for the elderly (as seen in the regression analyses). In particular, the distribution of costs among hospital admissions, outpatient care and drugs was relatively equal until the patients reached age 70–74, at which age almost 80% of the cost consisted of hospitalization. Older patients received fewer treatments (shorter cycles and/or less expensive chemotherapies) that were typically provided in the hospital setting rather than in the outpatient setting, which was less efficient.

**Fig. 2:**
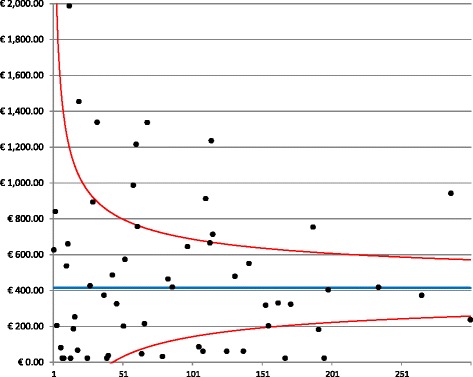
Funnel plot of diagnostic costs. The graph demonstrates the dispersion around the mean costs (blue line) for each provider (black dots) as a function of the hospital volume. The red lines represent the 95% confidence interval around the mean

With the aim of investigating how the complexity of hospital, measured by the volume of specific breast cancer procedures, might influence the costs per patient, funnel plot analyses were used to examine the wide range among hospitals. Figure 2 presents the funnel plot of the diagnostic costs in the treating hospitals in the Province of Milan. The vertical axis represents the mean cost, whereas the horizontal axis represents the volume of activity of each provider in terms of patients treated for breast cancer. Of the 71 hospitals, 28% fell outside the 95% funnel plot confidence limits. The figure demonstrates little dispersion around the mean cost (blue line).

**Fig. 3:**
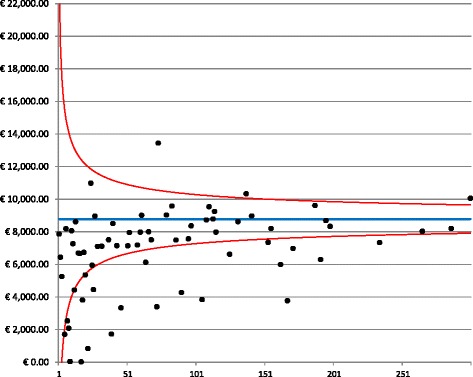
Funnel plot for treatment costs. The graph depicts the dispersion around the mean cost (blue line) for each provider (black dots) as a function of the hospital volume. The red lines represent the 95% confidence interval around the mean

The highest variability in costs was observed for the treatment costs, in which 32% of hospitals fell outside the 95% confidence interval (Fig. 3). In particular, small providers spent less for treatments than did providers with higher volumes of patients.

**Fig. 4:**
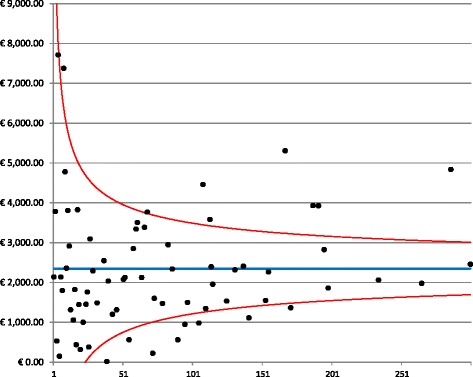
Funnel plot for follow-up costs. The graph depicts the dispersion around the mean costs (blue line) for each provider (black dots) as a function of the hospital volume. The red lines represent the 95% confidence interval around the mean

A funnel plot of the follow-up costs is presented in Fig. 4. The plot reveals a greater dispersion of costs compared with the previous mean cost, with 21% of hospitals falling outside the 95% confidence interval. Follow-up costs were collected over a period of two years, a period that represents a sufficient length of time to determine whether the patients complied with the standard follow-up. These results indicated that in many hospitals, more resources were devoted relative to the standard follow-up, which consists of visits and mammography only. In the over-spending hospitals, patients received an intensive followup regimen with scintigraphy and thoracic CAT (computerized axial tomography). These results confirmed a previous study that reported that intensive post-operative follow-ups are routinely employed in Milan hospitals, creating a burden on the financial assets of the hospitals without conferring a clear prognostic and therapeutic advantage [20].

The highest variability in costs was observed for the treatment costs, in which 32% of hospitals fell outside the 95% confidence interval (Fig. 4).

**Fig. 5:**
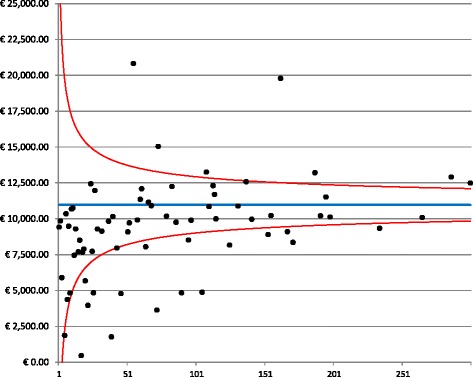
Funnel plot for the total cost. The graph depicts the dispersion around the mean costs (blue line) for each provider (black dots) as a function of the hospital volume. The red lines represent the 95% confidence interval around the mean

Finally, the funnel plot of the total direct medical costs (Fig. 5) revealed less intense care among small providers, which is consistent with the hypothesis of more expensive treatment and follow-up in cancer institutes and in general hospitals with high patient volumes.

## Discussion

In this study, which represents the first population-level study of its kind in Italy, we estimated all direct medical costs for the 6-month period before the diagnosis of breast cancer and the first two years after diagnosis. Patients were identified from the local cancer registry. The registry information on a defined population of incident cancer cases was linked to the longitudinal administrative records, thereby providing a basis for tracking patients and estimating the treatment costs.

We calculated the costs incurred by 12.580 individuals with breast cancer (the entire breast cancer population of the Province of Milan for 3.2 million inhabitants) thought to be directly related to the detection or treatment of the disease. The direct medical costs have been divided into diagnosis, treatment and follow-up costs. It is worth noting that these cumulative results are also of considerable interest to clinicians, managers and policy makers [13]. Moreover, this approach describes the complete costs of an “average” patient from diagnosis up to the first two years of follow-up, thus avoiding the problems of censored costs [21]. Patient age, tumor stage and employment level were identified as significant predictors of mean costs. Elderly patients were treated at a lower cost compared with younger patients due to less expensive treatments and fewer follow-up tests. High co-morbidities lead to lower costs, which is consistent with the reduction in chemotherapies and radiotherapies due to cardiovascular diseases.

The main analysis was to identify how the volume of activities (number of patients with breast cancer during the period of observation) might account for the different main costs by hospitals. To our knowledge, this objective has never been realized in any previous published study, particularly in Italy. A study of colorectal cancer calculated the patterns of care and costs in two regions using a three-phase disease approach that linked the regional hospital discharge form database and the cancer registry database. The study reported the mean cost by age and by stage but not by hospital volume activity [22]. Small hospitals offered less intense care than the largest hospitals, confirming that expensive treatment and follow-up are more common in cancer institutes and in general hospitals with high volume of patients.

Additionally, our study is the first to calculate the real cost for each hospital, including the estimation not only of the tariffs reimbursed to each hospital on a DRG basis but also the other relevant costs imputed on the total budget, plus the unit cost for each procedure (laboratory tests, visit, etc.) and for expensive drugs (biologics). An excess of procedures during the follow-up phase has been demonstrated, confirming the excessive utilization of eco marker, scintigraphy and PET (Positron Emission Tomography) (these tests are not recommended according to guidelines [23, 24]. This situation is similar to other countries in which the rising cost of cancer care is due, in part, to unnecessary use of health care [25].

A quite extensive literature investigated the relationship between socioeconomic variables and treatment patterns, variation in diagnostic investigations and treatments in cancer care [26, 27]. Our study found that socioeconomic variables (deprivation, education and job categories) are directly associated to the cost only in the follow up and not in the diagnostic and treatment phases. Indeed, during the follow-up there are less constraints for hospital physicians to provide a quite wide range of procedures, and these decisions might be influenced by the level of education and economic status of the patient.

One limitation of our study consisted of the secondary data analysis. Administrative data sources are widely used to derive measures of resource use; however, they do not necessarily convey accurate information about the economic costs of procedures and services. Another limitation was that given the relatively small cohort, our study was unable to prolong the observational period to the metastatic phase. Furthermore, because data about private expenditures for cancer care (out-of-pocket) are not available, we do know how the socioeconomic level of the patients might have influenced the utilization of public resources and whether a crowding effect might be present in some jurisdictions (hospital in the most affluent areas could exhibit lower utilization rates due to the tests and visits performed outside the public system). Finally, our study evaluated only direct medical costs and not indirect costs, such as lost productivity. Other work has indicated that indirect costs are substantial, accounting for well over 50% of the total cost of cancer [28–30].

## Conclusions

Our study represents an attempt to improve the capture and analysis of patient data for assessing disease burden. Furthermore, our study attempts to understand the resources needed to provide appropriate care, which is an ambitious goal for health policy makers [31].

The Italian system is facing the same problems of any other European countries in terms of budget constraints, lack of efficiency, partial adherence to guidelines, increasing number of cancer patients, increasing cost of cancer drugs, etc. Our study is giving a contribution in order to have a better understanding of breast cancer care, mainly from the resource utilization point of view.

Future analyses will examine various time frames or phases throughout the management of breast cancer. The methods used here will help future work on the cost of breast cancer and other types of cancer to improve our understanding of stage-based resources and funding.

## Abbreviations

AIC: Number issued by the Italian Drugs Agency for commercialization
ATC: Anatomical Therapeutic Chemicals
CAT: Computerized Axial Tomography
DRGs: Diagnosis-Related Groups
ER: Emergency Room
GLM: Generalized Linear Model
ICU: Intensive Care Unit
LHA: Local Health Authority
PET: Positron Emission Tomography
